# Syrian Adolescent Refugees: How Do They Cope During Their Stay in Refugee Camps?

**DOI:** 10.3389/fpsyg.2018.01258

**Published:** 2018-07-20

**Authors:** Orna Braun-Lewensohn, Khaled Al-Sayed

**Affiliations:** ^1^Conflict Management & Resolution Program, Department of Multidisciplinary Studies, Ben-Gurion University of the Negev, Beersheba, Israel; ^2^Kaye Academic College of Education, Beersheba, Israel

**Keywords:** adolescents, refugees, coping, psychological problems, refugee camp, post-traumatic stress symptoms

## Abstract

To date, more than 11 million Syrians have been forced from their homes due to the civil war in that country. However, little research has been done on adolescent Syrian refugees. This study aimed to fill that gap in the research literature by examining how adolescent Syrian refugees cope with the harsh situation of having fled from their homes. We explored how personal capital factors, sense of coherence (SOC), wishes, and expectations, as well as socio-demographic and situational factors, contribute to a variety of mental health and psychological problems, namely, internalizing and externalizing problems and post-traumatic stress symptoms. Data were gathered from 110 adolescents aged 13–18 of whom 50.9% were boys. Participants filled out self-report questionnaires that asked about demographics, exposure to war, appraisal of danger, receiving aid, SOC, wishes, and expectations. They also completed the Achenbach Youth Form. The results show that girls appraised their situation as more dangerous and reported more internalizing, externalizing, and post-traumatic stress symptoms. By contrast, boys reported more exposure to war experiences and stronger SOC. Younger adolescents reported stronger SOC, while older adolescents reported more psychological problems. The adolescents who had more recently arrived in the refugee camp were in better condition, thereby reporting stronger SOC, higher expectations, and fewer psychological problems. The amount of time spent in the refugee camp, gender, exposure to war situations, and appraisal of danger all contributed to the explained variance in the different psychological problems. However, once the personal resource SOC was entered into the model, it mediated the relationships between all of the socio-demographic and situational variables, on the one hand, and the examined psychological problems, on the other. The results are discussed based on the personal-capital model of salutogenesis and its relations with traumatic stress.

## Introduction

Since 2011, more than 250,000 Syrians have lost their lives in armed conflict and more than 11 million others have been forced from their homes due to the civil war and the penetration of ISIS forces into Syria (British Broadcasting Corporation, 2016). To date, the majority of those in need have sought refuge in neighboring countries or within Syria itself (European Union, 2018). However, very few studies have focused on Syrian refugees, specifically adolescent refugees and their recovery capital. The present study aims to fill this void in the research literature.

Based on the salutogenic model (Antonovsky, 1979, 1987), the present study aimed to explore the personal capital of adolescent refugees who have fled Syria to a European country. More specifically, we wanted to compare boys with girls and younger adolescents with older adolescents, as well as those who had lived in a refugee camp for more than 6 months (up to 2 years) with those who had recently arrived in the camp (1–6 months). We compared these subgroups in terms of several factors, specifically, mental health (post-traumatic stress, internalizing problems, and externalizing problems), personal capital (i.e., coping resources), exposure to the armed conflict, appraisal of danger in the war zone, and receiving aid from a variety of organizations. In addition, we also wanted to facilitate an understanding of the variables that could explain mental health among adolescent refugees.

### Adolescent Refugees

Refugees, especially adolescents exposed to and experiencing war, are at risk of developing psychological problems (Bean et al., 2007) because of their unique developmental stage. Their vulnerability is partially due to role confusion and transformation in responsibilities and identity (Arnett, 2000). However, studies of the mental health of refugees are inconclusive and show a great degree of variability. Furthermore, even studies that have shown high rates of psychological problems upon arrival to the refuge point to a reduction in symptoms over time (Sack et al., 1999). Moreover, many refugee youth do exceptionally well and have relatively good mental health. Factors that facilitate the ability to overcome the difficult situations and transitions faced by these adolescents include strong parental and social support, as well as personal and community resources, which aid in reducing negative emotional and behavioral outcomes (Braun-Lewensohn, 2015). Additionally, one of the strengths of adolescents is their adaptability, which can assist them as they adjust to their new environments (Weine et al., 1995).

### Exposure to Violence

Direct exposure to political violence refers to the individuals’ experience of rockets/bombs falling on and damaging their neighborhood and surroundings, as well as harm done to their acquaintances as a result of the war (Braun-Lewensohn et al., 2009b). This type of exposure to violence is likely to increase the risk of adverse psychological problems, such as post-traumatic stress and other internalizing problems, especially during the first stage of migration (Geltman et al., 2005; Montgomery, 2008). Results of studies regarding cumulative exposure to violent political events are inconclusive (Fazel et al., 2012). While some studies show associations between exposure, namely, the number of events and their intensity, and various psychological problems among refugee groups (Grgić et al., 2005; Derluyn and Broekaert, 2007; Eggerman and Panter-Brick, 2010), other studies have indicated that the number of events is not the most significant predictor of post-traumatic stress or other psychological problems (Berthold, 2000; Braun-Lewensohn et al., 2009a). As for socio-demographic variables such as gender and age, no differences were observed regarding this type of exposure (Braun-Lewensohn et al., 2009b).

### Appraisal of Danger

According to Lazarus and Folkman (1984), there are two kinds of appraisal: primary appraisal and secondary appraisal. While primary appraisal is the evaluation of the original threat in order to estimate the current threat, secondary appraisal is the assessment of the resources one has to deal with the stressor. The importance of appraisal lies in the fact that the evaluation of whether a situation represents a threat or a challenge determines the level of arousal and coping performance.

In the present study, we evaluated the appraisal of danger as primary appraisal. This kind of appraisal has been examined in various ways in a number of studies. Some studies in the context of political violence have defined it as subjective experiences of exposure to particular situations. These have included reports of peri-traumatic reactions (i.e., initial fears), worries about the safety of family members and friends (Pfefferbaum et al., 2002, 2003), and levels of self-reported fear (Laufer and Solomon, 2003; Solomon and Lavi, 2005), in addition to feelings of danger to oneself and one’s close and wider community/environment. Studies show that girls and older adolescents are more vulnerable and report more feelings of danger (Braun-Lewensohn et al., 2009b; Braun-Lewensohn, 2012). The overall results show that exposure to violence does not always correlate with more feelings of danger and worry. However, these feelings seem to be an independent predictor of a variety of mental health symptoms. Thus, the stronger the feelings of danger are, the higher the threat appraisal, and the more intense the mental health symptoms (Pfefferbaum et al., 2002, 2003; Solomon and Lavi, 2005; Braun-Lewensohn et al., 2009a).

### Receiving Aid

The Western idea behind humanitarian aid and support focuses mainly on material and social support, and there is some debate about whether that aid, which is funded by a variety of organizations with political agendas, actually benefits, and harms or does not affect the refugees who receive it (Almedon, 2004). Most studies in this domain lack empirical evaluations, but rather focus on the burnout of aid workers (e.g., Eriksson et al., 2009; Cardozo et al., 2012). Moreover, very little has been done to translate lessons learnt into actual policy and practice (Minear, 2002; Terry, 2002).

One study that tried to evaluate whether humanitarian aid mitigates or exacerbates the effects of war on stress reactions was based on two interviews and did not draw a clear conclusion. Additionally, despite the problematic idea of organizations driven by external interests, that work did not suggest relying solely on the refugees’ needs and priorities (Almedon, 2004). Thus, the present study will try to fill in this lacuna by examining aid from a variety of organizations, governments, and family or community members, and its relationships with mental health problems.

### Recovery Capital

Recovery capital includes resources accumulated over time and different life experiences. These resources include health, well-being, social relationships, family cohesion, and life satisfaction, among others (Dennis et al., 2007). Recovery capital includes social capital, physical capital, human capital, and cultural capital (Cloud and Granfield, 2008; White and Cloud, 2008; Lyons and Lurigio, 2010). Social capital refers to membership in a social group. This membership awards individual members of the group access to resources and benefits; however, it also imposes obligations to the group. It is an important construct during crises, since it allows for options, information, and support to emerge. Indeed, individuals who report strong social capital have an easier time recovering from crises and maintaining well-being (Antonovsky, 1987). Physical capital includes more concrete and tangible resources such as financial capabilities including income, savings, investments, and other assets. Cultural capital is a function of the values, beliefs, and norms of one’s group. These perceptions enable the individual to maximize opportunities within his/her cultural group (Cloud and Granfield, 2008). Finally, human capital is the individual’s set of skills, behavioral characteristics, and personal styles, including personal relationships (Caspi et al., 1998; Hook and Courtney, 2011). The present study will evaluate three types of human and personal capital, namely, sense of coherence (SOC), wishes, and expectations, which are components of hope.

#### Sense of Coherence

Antonovsky (1979) suggested a continuum model to conceptualize stress research. Accordingly, people have resources that can help them conceptualize the world as organized, rational, consistent, and understandable. SOC, the core construct of this model, is a form of personal capital that represents the internal and external resources one believes are available for him/her to cope with stressors. Additionally, the motivation to cope and the commitment to emotionally invest in the coping process are inherent in the SOC concept. The salutogenic model suggests that an individual with a strong SOC is less likely than one with a weak SOC to perceive many stressful situations as threatening and, therefore, anxiety-provoking. Given their tendency to perceive the world as meaningful and manageable, individuals with a strong SOC will be less likely to feel threatened by experiences of political violence and the experience of becoming a refugee, and are less vulnerable when they have had those experiences. Like other types of personal capital, SOC evolves during adolescence as part of the developmental process. As it requires complex cognitive and emotional skills, SOC becomes stronger in late adolescence (Nilsson et al., 2010). Regarding gender, most studies have shown men to have stronger SOC than women, including during adolescence (Moksnes et al., 2012). Finally, one of the most important criteria for the development of SOC is the stability of the community, as such stability helps adolescents to perceive the world around them as predictable and manageable (Antonovsky, 1987).

#### Wishes and Expectations

Wishes and expectations are affective and cognitive aspects of hope that are considered important assets and a form of personal capital, especially in times of threat (Lazarus, 1966; Staats, 1989). When an individual experiences threat and deprivation, s/he considers the future and wishes for something in the future that does not exist at present. Threat and deprivation stimulate the need for these wishes for stability (Staats and Partlo, 1993). The cognitive component is important for the development of hope and positive outcomes, since it leads to problem-focused thoughts and actions, thereby reducing the possibility of despair through appropriate and fit responses (Staats, 1991; Staats and Partlo, 1993).

Despite the difficulties and barriers to assimilation and acculturation in their new environments, adolescent refugees have been reported to express high expectations for a bright future (Nunn et al., 2014). Both genders report approximately the same amount of hope (Greene and DeBacker, 2004). Wishes and expectations, as representations of hope for the future, have been highlighted as mediators of stressful life events in various cultures (Braun-Lewensohn and Sagy, 2011). Moreover, it has been noted that wishes and expectations are extremely important for adolescents whose futures may be unstable and unpredictable, including refugee youth (Nalkur, 2009).

### Psychological Problems Among Adolescent Refugees

War experiences have been linked with a variety of psychopathologies and psychological problems and symptoms among adolescent refugees (Lustig et al., 2004). Indeed, some studies report refugee youth as having severe symptoms of anxiety, depression, and post-traumatic stress (Derluyn and Broekaert, 2007). However, other studies have found adolescents refugees to be resilient, despite their difficult life experiences and exposure to violence (Lustig et al., 2004).

As for socio-demographic variables in this context, the stress literature has considered age to be a protective factor against maladaptive outcomes (Fazel et al., 2012). However, contrary to the general literature, during long periods of political violence, older adolescents have been found to report more stress reactions than younger adolescents (Braun-Lewensohn et al., 2009a). As for gender, girls seem to be more vulnerable to internalizing problems and post-traumatic symptoms; whereas boys report more externalization (Derluyn and Broekaert, 2007; Braun-Lewensohn, 2010).

### The Current Study

The current study aims to fill a gap in the research literature which lack studies on adolescent refugees and their recovery capital. More specifically, studies are inconclusive in their results regarding adolescent refugees’ mental health and fail to address resources which aid adolescents to overcome the difficult situation of being refugees and fleeing war and atrocities. In accordance with the literature review, the following research questions and hypotheses were formulated:

(1) Are there differences between genders, age groups, and participants that have spent more vs. less time in a refugee camp, in terms of the various situational factors of exposure to war events, feelings/appraisal of danger and receiving aid from a variety of individuals, organizations, and governments? Are there differences relating to the coping resources of SOC and wishes and expectations, or to psychological outcomes (i.e., internalizing, externalizing, and posttraumatic stress symptoms)?
(1a) Girls will report more internalizing of problems and post-traumatic symptoms, as well as stronger appraisal of danger, whereas boys will report more externalizing of problems and stronger SOC (Braun-Lewensohn et al., 2009a; Braun-Lewensohn, 2010; Moksnes et al., 2012). No gender differences are expected in terms of objective exposure, wishes, or expectations (Greene and DeBacker, 2004).
(1b) Older adolescents will report more psychological problems and higher appraisals of danger, whereas we do not expect to find any differences between older and younger adolescents in terms of the other variables (Braun-Lewensohn et al., 2009a).
(1c) No hypothesis was formulated regarding the effect of time spent in the refugee camp, since there is not enough literature in this area upon which to base any such hypothesis.
(2) What are the relationships between the study variables of exposure to war events, appraisal of danger, receiving aid, SOC, wishes, expectations, internalizing, externalizing, and post-traumatic stress symptoms? Overall, exposure to war events and high appraisal of danger are expected to positively correlate with stress symptoms and negatively correlate with SOC, wishes, and expectations, which, in turn, are expected to negatively correlate with stress symptoms (Braun-Lewensohn et al., 2009a; Braun-Lewensohn and Sagy, 2011).
(3) Finally, we evaluated a model in which gender; age; time spent in the refugee camp; exposure to political violence; feelings of danger; aid from organizations, governments, family, and/or community members; and coping resources were entered as predictors of the psychological problems of internalizing, externalizing, and post-traumatic stress.

## Materials and Methods

### Participants

One hundred and ten Syrian refugees living in a refugee camp in Europe participated in the study. No inclusion or exclusion criteria were used apart from age (12–18) and a convenience sampling was used. The mean age of the participants was 15.48 (*SD* = 1.35). Females accounted for 49.1% of the sample. As for ethnic groups, Kurds accounted for 20% of the sample (22 participants), Sunnis for 70% (77 participants), and Yazidis for 10% (11 participants). Forty-eight participants (43.6%) had lived in the refugee camp for up to 6 months, while 62 (56.4%) had lived in the camp between 6 months and 2 years.

### Procedures

Data were collected by self-reported questionnaires during May–July 2017 in a refugee camp in Europe. Prior to administration of the questionnaires, the research was evaluated and approved by the university (Ben-Gurion University of the Negev) department’s (Conflict Management & Resolution) ethics committee (Number 2017-04). All ethical standards were maintained accordingly. With accordance to the requirements of the ethics committee, passive consent was obtained from the parents of the participating youth. Letters regarding the study were sent to the parents or guardians of the children in their native tongue (Arabic). Parents who did not want their child to participate in the study signed a form indicating their wish. Twelve parents signed that form and, therefore, their children did not participate in the study. In addition, approximately 14 participants were approached when they participated in a workshop with their parents. In that case, the parents and children received an oral explanation of the study and questionnaires, and the parents gave permission for their children to participate on the spot. All participants were informed that the researchers were interested in their experiences, participation was voluntary and anonymity was emphasized. The questionnaires were translated into Arabic by an Arabic language teacher and then back-translated into Hebrew to ensure the accuracy of the translation.

### Measures

Demographic characteristics included questions regarding the participants’ gender, age, and ethnicity and when they had first entered the refugee camp.

Exposure to violent political events was assessed using five yes (1)/no (0) questions that referred to whether one’s community had been attacked by rockets/bombs; whether someone the individual knows had been hurt as result of the war; whether a relative had been hurt as a result of the war; whether the individual him/herself had been hurt as result of the war; and whether the individual’s home had been damaged as result of the war. The answers to the different questions were summed to calculate an index with a potential range of 0–5 (*M* = 3.33, *SD* = 1.17).

Appraisal of danger was assessed using an index of four questions, each of which were answered using a 5-point Likert scale (1 – *not at all*; 5 – *very much*). Questions related to how dangerous the situation in Syria was for the study participant, his/her family, his/her friends, and civilians in Syria. Answers were summed up to create an index ranging from 4 to 20 (*M* = 18.97, *SD* = 1.17).

The variable “receiving aid” was assessed by six questions each answered using a 5-point Likert scale (1 – *not at all*; 5 – *very much*). Questions related to receiving aid from family members, Muslim organizations, aid organizations, governments in Europe, and the United Nations. A mean score was calculated to create an index with a range of 1–5 (*M* = 4.11, *SD* = 0.72).

Sense of coherence (Antonovsky, 1987) was measured using a series of semantic differential items on a 7-point Likert-type scale, with anchoring phrases at each end. High scores indicated a strong SOC. An account of the development of the SOC scale and its psychometric properties, showing it to be reliable and reasonably valid, appears in Antonovsky’s (1987, 1993) writings. In this study, SOC was measured by the short-form scale consisting of 13 items, which was found to be highly correlated to the original long version (Antonovsky, 1993). The scale includes such items as: “Doing the things you do every day is” with answers ranging from (1) “a source of pain and boredom” to (7) “a source of deep pleasure and satisfaction.” In the present study, the Cronbach’s alpha coefficient was 0.82.

Hope was construed as the interaction of wishes and expectations, including aspects related to self and aspects related to others or to broad global concerns (Staats, 1989). Some items such as “to be competent” and “to be happy” reflect one’s hope for oneself while other items reflect hope regarding global issues, such as “peace in the world” and “justice in the world.” Participants were asked to independently rate the extent to which they would wish for a particular event to occur and the extent to which they would expect that event to occur. Responses were rated on a scale of 0 (*not at all*) to 5 (*very much*). The multiplication of the Wish value by the Expect value generated the measure of hope. Due to the negative correlation between the Wishes scale and the Expectations scale in the present study, each of these two components of hope was treated as a separate variable. The Cronbach’s alpha coefficient for Wishes was α = 0.88 and the Cronbach’s alpha coefficient for Expectations was α = 0.98.

The Achenbach Youth Self-Report for Ages 11–18 (Hebrew version; Achenbach, 1991; Achenbach and Rescorla, 2001) measures a broad range of behavioral and emotional problems through 112 items, yielding a total problem score along with two broad-band scales (internalizing and externalizing), eight subscales, and six DSM scales. This instrument has been shown to have good internal consistency, test–retest reliability (0.87), and content validity (Achenbach and Rescorla, 2001). The Cronbach’s alpha values for the different scales in the present study were sufficient and ranged from 0.73 to 0.85.

### Data Analysis

Statistical analyses were conducted with the statistical software SPSS Version 25. A significance level (alpha) of *p* < 0.05 was chosen. First, frequencies and percentages of the sample’s demographic characteristics were explored. Second, we ran *t*-tests for independent samples to evaluate the effects of gender, age groups, and time spent in the refugee camp on the different study’s variables. Third, Pearson correlations were calculated to examine the relationships between exposure, appraisal of danger, receiving help from an aid organization, different human capital variables, and the outcome variables of internalizing and externalizing problems and post-traumatic stress symptoms. Fourth, hierarchal regressions were calculated to explain the variance of internalizing, externalizing, and PTSS by the different study’s variables. Finally, the PROCESS component of SPSS was used to evaluate whether SOC mediated the relationships between the different demographic or situational variables and the outcome scales.

## Results

Overall, our results show that the study participants scored on the higher ends of all of the psychological problem scales. Regarding the frequency of post-traumatic stress, while the minimum report was 0.93 the maximum was 2.00 with an average level of 1.57 (*SD* = 0.26). This means that participants in this study reported post-traumatic symptoms on the higher end of the scale. Moreover, their internalizing and externalizing of problems and their exposure to war experiences and appraisal of danger were also on the higher ends of the scales. As for personal capital and resilience factors, while their SOC and expectations were very low, their wishes were very big, expressing a large gap between the cognitive and emotional components of hope.

To answer our first research question, *t*-tests for independent groups were run to evaluate differences among genders, age groups, and time of arrival groups. The results support the hypothesis and show that the girls reported more appraisal of danger, internalizing, and post-traumatic stress symptoms (**Tables 1**–**3**). The boys reported more exposure to war experiences and stronger SOC. Contrary to our hypothesis, girls also reported more externalizing of problems.

**Table 1:** Differences in the study variables among boys and girls.

Variable (range)	Boys		Girls		*t*	Cohen’s *d*
				
	*M*	*SD*	*M*	*SD*		
SOC (1–7)	2.93	0.55	2.49	0.81	3.31**	0.63
Wishes (1–5)	4.27	0.56	4.35	0.36	-0.83	-0.16
Expectations (1–5)	1.27	1.39	1.00	1.10	1.12	0.22
Receiving aid (1–5)	4.18	0.70	4.03	0.72	1.03	0.21
Appraisal of danger (4–20)	18.62	1.29	19.33	0.91	-3.34**	-0.63
Exposure index (0–5)	3.64	0.94	3.02	1.30	2.80**	0.54
Internalizing (0–2)	1.35	0.16	1.63	0.19	-8.16***	-1.59
Externalizing (0–2)	1.04	0.25	1.17	0.31	-2.29*	-0.46
Post-traumatic stress (0–2)	1.43	0.20	1.71	0.23	-7.03***	1.21


*^∗∗∗^p < 0.001; ^∗∗^p < 0.01; ^∗^p < 0.05. Boys (n = 56); girls (n = 54).*

**Table 2:** Differences in the study variables among younger and older adolescents.

Variable (range)	13–15 years old		16–18 years old		*t*	Cohen’s *d*
				
	*M*	*SD*	*M*	*SD*		
SOC (1–7)	2.86	0.70	2.56	0.70	2.34*	0.42
Wishes (1–5)	4.28	0.59	4.35	0.33	-0.72	-0.15
Expectations (1–5)	1.25	1.16	1.04	1.35	0.86	0.16
Receiving aid (1–5)	4.24	0.67	4.01	0.74	1.69	0.32
Appraisal of danger (4–20)	19.0	0.91	19.00	1.31	0.00	0
Exposure index (0–5)	3.36	1.24	3.31	1.13	0.19	0.04
Internalizing (0–2)	1.44	0.20	1.53	0.23	-2.26*	-0.41
Externalizing (0–2)	1.05	0.29	1.15	0.27	-1.93	-0.36
Post-traumatic stress (0–2)	1.54	0.25	1.60	0.26	-1.33	-0.23


*^∗^p < 0.05. 13- to 15-year-olds (n = 51); 16- to 18-year-olds (n = 58).*

**Table 3:** Differences in the study variables among adolescents who had been in the camp for shorter and longer periods.

Variable (range)	Up to 6 months		6 months–2 years		*t*	Cohen’s *d*
				
	*M*	*SD*	*M*	*SD*		
SOC (1–7)	2.93	0.62	2.54	0.74	2.94**	0.57
Wishes (1–5)	4.23	0.60	4.34	0.33	-1.72	-0.22
Expectations (1–5)	1.53	1.46	0.84	1.00	2.80**	0.55
Receiving aid (1–5)	4.15	0.66	4.08	0.75	0.51	0.10
Appraisal of danger (4–20)	18.46	1.24	19.49	0.85	-4.41***	-0.97
Exposure index (0–5)	3.27	0.97	3.38	1.31	-0.47	-0.09
Internalizing (0–2)	1.37	0.19	1.58	0.19	-5.45***	-1.10
Externalizing (0–2)	0.96	0.21	1.21	0.27	-5.40***	-1.03
Post-traumatic stress (0–2)	1.45	0.23	1.66	0.23	-4.75***	-0.91


*^∗∗∗^p < 0.001; ^∗∗^p < 0.01. Up to 6 months (n = 47); 6 months to 2 years (n = 62).*

The effects of age were much more minor than the effects of gender and not always in the expected direction. Older adolescents reported weaker SOC and more internalizing of problems.

Finally, the newer arrivals (in the camp for no more than 6 months) were in better condition than those who had been living in the refugee camp for a longer period. The new arrivals reported stronger SOC, higher expectations, and fewer psychological problems.

The second question was answered by means of Pearson correlations (**Table 4**), and the results partially support the hypothesis. Results show that while appraisal of danger was positively related to all psychological problems, as expected by the hypothesis, exposure to war experiences and reports of receiving aid were not related to any problem. However, the more aid an adolescent received, the greater expectations for the future s/he reported. Moreover, the more perceived (appraised) danger and the more s/he had been exposed to war experiences, the weaker SOC s/he reported. It should also be noted that, in contrast to most studies, we observed a negative correlation between wishes and expectations. Finally, as for the coping resources relating to the psychological problems, while SOC was negatively related to all problems, expectations were related only to externalizing problems and wishes were not related to any psychological problem.

**Table 4:** Pearson correlations between the study variables.

	1	2	3	4	5	6	7	8	9
1. Appraisal of danger	–	0.06	0.02	-0.38***	-0.01	0.19*	0.44***	0.26**	0.36***
2. Exposure index		–	0.23*	-0.25*	0.00	0.14	-0.05	0.16	-0.05
3. Receiving aid			–	0.09	0.11	0.24*	-0.08	-0.05	-0.05
4. SOC				–	0.01	0.00	-0.49***	-0.44***	-0.46***
5. Wishes					–	-0.29**	0.15	0.11	0.16
6. Expectations						–	-0.04	-0.40***	-0.06
7. Internalizing							–	0.52***	0.83***
8. Externalizing								–	0.48***
9. PTSS									–


*^∗∗∗^*p* < 0.001; ^∗∗^*p* < 0.01; ^∗^p < 0.05.*

The last question explored a model in which the socio-demographic variables of gender, age, and time spent in the camp were entered in the first step; situational variables of appraisal of danger, exposure to war experience, and receiving aid were entered in the second step and SOC, wishes, and expectations were entered in the last step, to explain internalizing, externalizing, and post-traumatic stress problems (**Table 5**). Additionally, we evaluated whether SOC mediated the relationships between the socio-demographic and the situational variables, on the one hand, and psychological problems, on the other.

**Table 5:** Results of hierarchical multiple-regression analysis predicting psychological problems.

	Post-traumatic stress					Externalizing problems					Internalizing problems				
			
	*R*^2^	*B*	β	*SE*	*t*	*R*^2^	*B*	β	*SE*	*t*	*R*^2^	*B*	β	*SE*	*t*
**Step 1**	0.54					0.24					0.42
Gender		0.25	0.57	0.03	8.09***		0.06	0.11	0.05	1.26		0.26	0.52	0.04	6.57***
Age		0.07	0.15	0.03	2.16*		0.07	0.13	0.05	1.43		0.04	0.08	0.04	1.01
Time spent in camp		0.14	0.30	0.03	4.27***		0.23	0.41	0.05	4.46***		0.14	0.26	0.04	3.32**
**Step 2**	0.03					0.04					0.01
Gender		0.25	0.56	0.03	7.56***		0.09	0.16	0.05	1.67		0.27	0.53	0.04	6.24***
Age		0.07	0.16	0.03	2.36**		0.07	0.13	0.05	1.40		0.05	0.09	0.04	1.19
Time spent in camp		0.11	0.24	0.03	3.15**		0.21	0.37	0.06	3.84***		0.11	0.22	0.04	2.60*
Exposure index		0.01	0.07	0.01	1.03		0.05	0.20	0.02	2.13*		0.01	0.07	0.02	0.81
Appraisal of danger		0.03	0.16	0.01	2.18*		0.01	0.02	0.02	0.25		0.02	0.09	0.02	1.08
Help		0.00	0.01	0.02	0.13		-0.02	-0.06	0.04	-0.67		0.01	0.02	0.03	0.28
**Step 3**	0.04					0.19					0.05
Gender		0.22	0.50	0.03	6.54***		0.01	0.03	0.05	0.29		0.23	0.45	0.04	5.14***
Age		0.04	0.10	0.03	1.45		0.04	0.07	0.05	0.85		0.01	0.02	0.04	0.32
Time spent in camp		0.12	0.27	0.03	3.38**		0.11	0.20	0.05	2.14*		0.12	0.23	0.05	2.56*
Exposure index		0.00	0.00	0.01	-0.04		0.03	0.11	0.02	1.31		0.00	-0.02	0.02	-0.22
Appraisal of danger		0.01	0.07	0.01	0.92		0.02	0.09	0.02	1.01		0.00	0.01	0.02	0.13
Help		-0.01	-0.02	0.02	-0.26		0.02	0.05	0.03	0.64		0.00	0.01	0.03	0.08
Sense of coherence		-0.06	-0.20	0.03	-2.52*		-0.13	-0.32	0.04	-3.42**		-0.09	-0.24	0.03	-2.55*
Wishes		0.06	0.12	0.03	1.76		-0.02	-0.04	0.05	-0.50		0.06	0.12	0.04	1.52
Expectations		0.03	0.15	0.01	1.89		-0.09	-0.40	0.02	-4.39***		0.02	0.10	0.02	1.15


*^∗∗∗^*p* < 0.001; ^∗∗^*p* < 0.01; ^∗^*p* < 0.05.*

The results show that for all of the psychological problems, the socio-demographic variables explained the most of the variance in internalizing (54%), externalizing (24%), and post-traumatic stress (42%). Whereas gender, age, and time spent in the camp played a major role in explaining internalizing problems, only gender and time spent in the camp were important for the explanation of post-traumatic stress. By contrast, only time spent in the camp played a significant role in explaining externalizing problems. As for the situational variables, they explained only a very limited amount of the observed variance. For internalizing problems, the only significant explanatory variable was appraisal of danger (3%) and for externalizing problems, the only significant explanatory variable was objective exposure (4%). The personal resource of SOC significantly explained the variance in all psychological problems, whereas expectations significantly explained the variance only in externalizing problems.

Finally, we used the PROCESS application in SPSS (Preacher and Hayes, 2004) to evaluate the indirect effect of the demographic or situational variables on the different psychological outcomes via SOC.

For externalizing problems, we found two significant models. The first model, included the independent variable exposure and explained 6% of the variance [*F*_(1,103)_ = 6.65, *p* < 0.05]. The standardized indirect effect of exposure on externalizing was as follows: bootstrapping 0.02:0.22, effect = 0.12. A significant model was also found for the independent variable of time spent in the camp. That model explained 7% of the observed variance [*F*_(1,107)_ = 8.61, *p* < 0.01] with a standardized indirect effect as follows: bootstrapping 0.05:0.35, effect = 0.18.

For internalizing problems, several significant models were found. The model in which gender was the independent variable explained 9% of the variance [*F*_(1,108)_ = 11.07, *p* < 0.01]. The standardized indirect effect of gender on internalizing was as follows: bootstrapping 0.07:0.36, effect = 0.20. When appraisal of danger was entered as an independent variable, the model explained 14% of the variance [*F*_(1,108)_ = 18.24, *p* < 0.001]. The standardized indirect effect of appraisal of danger on internalizing was as follows: bootstrapping 0.07:0.23, effect = 0.14. When age was entered as an independent variable, the model explained 5% of the variance [*F*_(1,108)_ = 6.08, *p* < 0.05]. The standardized indirect effect of age on internalizing was as follows: bootstrapping 0.04:0.21, effect = 11. When time of arrival was entered as an independent variable, the model explained 7% of the variance [*F*_(1,108)_ = 8.61, *p* < 0.01]. The standardized indirect effect of time of arrival on internalizing was as follows: bootstrapping 0.07:0.36, effect = 0.21.

Finally, for post-traumatic stress, we also found two significant models. When gender was entered as an independent variable, the model explained 9% of the variance in post-traumatic stress [*F*_(1,108)_ = 11.07, *p* < 0.01]. The standardized indirect effect of gender on post-traumatic stress was as follows: bootstrapping 0.06:0.34, effect = 0.19. When time spent in the camp was entered as an independent variable, the model explained 7% of the variance in post-traumatic stress [*F*_(1,107)_ = 8.61, *p* < 0.01]. The standardized indirect effect of time spent in the camp on post-traumatic stress was as follows: bootstrapping 0.05:0.36, effect = 0.19.

## Discussion

The ongoing civil war in Syria has forced millions of Syrians to flee their homes and head to other countries. In this study, we aimed to examine the personal human capital of adolescent Syrian refugees and the ways this capital helps them to cope with this harsh situation, as research in this area is lacking. We explored three main personal human capital factors: SOC, wishes, and expectations. We also evaluated the roles of socio-demographic and situational factors and their contributions to a variety of mental health and psychological problems.

Overall, our data support the fact that refugee adolescents are vulnerable, which means that their personal human capital is quite weak while their psychological problems and post-traumatic stress symptoms are very prevalent. Our results were similar to those other studies that have addressed political violence, in that we also found that girls are more fragile, exhibit more psychological problems and post-traumatic stress symptoms, and also appraise the situation as more dangerous (Braun-Lewensohn, 2010), while boys report more resiliency and stronger SOC (Moksnes et al., 2012). These results show that, although the gaps between boys and girls and men and women have been reduced in different Western countries, in more traditional societies and during times of war, adolescent boys are still in better condition than girls.

Our study also continues a line of studies that have claimed that a long period of war is more destructive for older adolescents who experience the war and its consequences for a longer period of time. We also found that the surveyed older adolescents were in worse condition than younger adolescents, thereby reporting more psychological problems and weaker SOC.

Time spent in the refugee camp was the most significant socio-demographic factor in this study. The significance of this factor had not been noted previously. Our results sadly show that the longer one stays in a refugee camp, the more severe psychological problem or post-traumatic stress symptoms s/he reports, and the more depleted and weak his/her SOC and expectations. This means that once the adolescent has fled from a war, s/he recruits his/her resources and recovery capital, human or cultural, to cope with the new situation. However, as time passes and the adolescents see no change, but rather continue to live at the refugee camp for a long period of time with no other options, they becomes less resilient, their resources (human, social, and cultural capital are depleted), and they are also less cognitively hopeful.

When we evaluated the relationships between the different variables, we found that most of the variables were related to each other in the expected way. Thus, appraisal of danger and SOC were the most meaningful in their relationships with the psychological problems. While stronger SOC meant more human capital and resiliency, the more one appraised his/her situation as dangerous, the more psychological problems or post-traumatic stress s/he reported (e.g., Eriksson and Lindström, 2007).

The surprising results in this study involved the relationships between the emotional and the cognitive components of hope, namely, wishes and expectations. When constructing the variable of hope, Staats (1989) related to wishes and expectations as complementing different aspects of the same construct. Moreover, she claimed that in threatening times, hope is strengthened (Staats and Partlo, 1993), since one hopes for something that one lacks. However, in this study, we found a negative correlation between these components of hope. That is, the greater the wishes, the lower the expectations. This indicates that, in spite of the fact that these adolescents emotionally wish and hope for a better future, when they evaluate the situation cognitively, they become hopeless and understand that their future is unlikely to be better than their present situation.

When assessing the entire model, we found that although the socio-demographic factors of gender and time spent in the refugee camp play important roles in explaining a variety of stress reactions and psychological problems, once the personal human resource of SOC enters the equation, their role decreases and even disappears. This is also similar to the different situational factors of exposure to war events and appraisal of danger. Also with regard to these variables, once the personal human resource of SOC is present, it becomes the most significant and important factor in reducing the various psychological problems. These results corroborate previous studies that have addressed adolescents’ coping with stress and political violence and demonstrated the important role of SOC in various cultural contexts (e.g., Braun-Lewensohn and Sagy, 2011). As for the other personal human resources of wishes and expectations, their roles in reducing psychological problems were less apparent. While wishes explained none of the psychological problems, expectations explained only externalizing problems. Thus, the higher the expectations, the fewer externalizing problems were reported, meaning that when one cognitively expects and hopes for a better future, s/he is less vulnerable to acting out and exhibiting anger via actions.

### Practical Implications

The results of the present study have some practical implications. First, it is very important to strengthen the SOC of adolescent refugees, in order to enable them to better cope with a variety of stressful situations. It is important that adolescents be included as integral parts of societal processes and feel that they have real potential to influence decisions regarding their lives. These interventions should take place in schools (a natural environment for development of human, social, and cultural capital) to facilitate better coping in the face of a variety of stressful situations. Moreover, workshops for parents with adolescents should be introduced to this group. The collectivistic nature of Arab society and the willingness of adolescents in this society to approach their parents and other significant adults for help when stressful situations occur make such workshops particularly appropriate for this population. Such workshops could raise the parents’ awareness of their role in such situations.

On the policy level, since the expectations of these adolescents are very low and decrease the longer they stay in the refugee camp, policy makers, and governments should consider moving these youngsters to permanent residences, in order to give them some hope and a better future.

### Study Limitations

Several limitations should be acknowledged. First, since all of the data were collected via self-report questionnaires, the extent to which adolescents’ experiences of mental health difficulties converge with external observations, such as parental, teacher, and clinical reports, remains to be investigated. Second, in the absence of any information about the participating adolescents’ psychological problems prior to the study period, we cannot state with certainty whether or not the observed outcomes are due solely to the impact of exposure to war and the refugee experience. Finally, a potential degree of sample bias cannot be ruled out as we investigated a relatively small sample, which was not a representative sample of adolescent Syrian refugees.

## Conclusion

Against the background of the civil war and the penetration of ISIS into Syria, the present study examined the role of the personal human resources of SOC, wishes and expectations (emotional and cognitive components of hope for better future) in reducing a variety of psychological problems among adolescents who had been forced from their homes in Syria. The participating adolescents had been residing in a refugee camp in Europe for a few weeks to 2 years. Our study showed that girls, older adolescents and those who have resided in the camp for longer periods of time are more vulnerable, exhibit more psychological problems, and possess less personal capital. Moreover, our results showed that the personal resource of SOC plays the most important role in explaining all of the examined psychological problems, and also mediates the relationships between a variety socio-demographic factors and exposure to war experience, on the one hand, and appraisal of danger, on the other.

Future studies should more thoroughly examine the role of the different kinds of aid received (e.g., humanitarian organizations, governments, family members, and community members), since incorporating all varieties of aids into one variable might mask any differential effects of different types of aid. Since additional traumatic exposure in the refugee camps has been found to account for much of the distress observed among adult refugees (Hoffman et al., 2018), that issue should also be examined among adolescent refugees.

## Author Contributions

OB-L was responsible for conceptualizing the paper, running the analysis, and writing the manuscript. KA-S was involved in conceptualizing the paper and data collection.

## Conflict of Interest Statement

The authors declare that the research was conducted in the absence of any commercial or financial relationships that could be construed as a potential conflict of interest. The reviewer AS and handling Editor declared their shared affiliation.
